# The Effect of 24/7, Digital-First, NHS Primary Care on Acute Hospital Spending: Retrospective Observational Analysis

**DOI:** 10.2196/24917

**Published:** 2021-07-22

**Authors:** Sam Winward, Tejal Patel, Mazin Al-Saffar, Matthew Noble

**Affiliations:** 1 Babylon Health London United Kingdom

**Keywords:** primary health care, family practice, general practice, cost, cost analysis, telemedicine, digital technology, digital health, digital care, virtual care, hospital, retrospective, observational, cohort, finance, economics, health services research

## Abstract

**Background:**

Digital health has the potential to revolutionize health care by improving accessibility, patient experience, outcomes, productivity, safety, and cost efficiency. In England, the NHS (National Health Service) Long Term Plan promised the right to access digital-first primary care by March 31, 2024. However, there are few global, fully digital-first providers and limited research into their effects on cost from a health system perspective.

**Objective:**

The aim of this study was to evaluate the impact of highly accessible, digital-first primary care on acute hospital spending.

**Methods:**

A retrospective, observational analysis compared acute hospital spending on patients registered to a 24/7, digital-first model of NHS primary care with that on patients registered to all other practices in North West London Collaboration of Clinical Commissioning Groups. Acute hospital spending data per practice were obtained under a freedom of information request. Three versions of NHS techniques designed to fairly allocate funding according to need were used to standardize or “weight” the practice populations; hence, there are 3 results for each year. The weighting adjusted the populations for characteristics that impact health care spending, such as age, sex, and deprivation. The total spending was divided by the number of standardized or weighted patients to give the spending per weighted patient, which was used to compare the 2 groups in the NHS financial years (FY) 2018-2019 (FY18/19) and 2019-2020 (FY19/20). FY18/19 costs were adjusted for inflation, so they were comparable with the values of FY19/20.

**Results:**

The NHS spending on acute hospital care for 2.43 million and 2.54 million people (FY18/19 and FY19/20) across 358 practices and 49 primary care networks was £1.6 billion and £1.65 billion (a currency exchange rate of £1=US $1.38 is applicable), respectively. The spending on acute care per weighted patient for Babylon GP at Hand members was 12%, 31%, and 54% (£93, *P*=.047; £223, *P*<.001; and £389, *P*<.001) lower than the regional average in FY18/19 for the 3 weighting methodologies used. In FY19/20, it was 15%, 35%, and 51% (£114, *P*=.006; £246, *P*<.001; and £362, *P*<.001) lower. This amounted to lower costs for the Babylon GP at Hand population of £1.37, £4.40 million, and £11.6 million, respectively, in FY18/19; and £3.26 million, £9.54 million, and £18.8 million, respectively, in FY19/20.

**Conclusions:**

Patients with access to 24/7, digital-first primary care incurred significantly lower acute hospital costs.

## Introduction

Health systems across the world are experiencing rising health care costs as a percentage of gross domestic product (GDP) [1]. As the global population continues to age [2], this is expected to worsen. Despite the associated increase in health outcomes, technological improvement is considered a further core driver of health care cost growth [3]. Resultantly, health systems rigorously assess the cost-benefit of such technologies before implementation. That same rigor has not been consistently applied to digital health solutions; hence, there is increasing concern that their proliferation is outpacing their monitoring and evaluation [4-7].

The COVID-19 pandemic has catalyzed the adoption and growth of digital technologies [8-11], intensifying the need to understand their impact. In particular, the use of telehealth, defined as the *“*delivery of healthcare services, where patients and providers are separated by distance” [12], has surged. As the world emerges from the pandemic, systems must begin to plan which technologies will form part of the new norm.

The capability of telehealth is well understood in terms of its potential to increase accessibility and patient satisfaction [13-17], increase efficiency [18,19], and improve clinical outcomes [20-22] while remaining safe [16]. The evidence is less clear on its cost-effectiveness. Several large-scale reviews concluded that the majority of interventions are cost-effective [23-25], while others are uncertain [19,26,27], including a Cochrane systematic review that stated the cost-benefit of telemedicine for a health system is unclear [26]. The contrasting nature of findings is in part related to the variety of evaluation methodologies and cost resources assessed; for example, one review stated that the predominant reason for cost savings was reduced travel costs [19]. Generally, telehealth cost-effectiveness studies are limited to a single clinical specialty or service modality but importantly do not consider the perspective of the health system [19]. This can lead to inconclusive assessments; as Rahimi [28] highlights, health systems are often in a state of disequilibrium, and new services can address unmet demand, which can cause net increases in expenditure.

One way to overcome this is to assess “all-cause” health care spending, as two recent digital health studies have done [29,30]. Both studies examined digital care management solutions that leverage proactive approaches and must be patient-focussed to succeed. As a result, they have higher potential to deliver savings to a health care system than do simpler telehealth-as-a-service solutions. Such offerings are most commonly a blend of telehealth, eHealth, and mobile health solutions but generally address a single condition.

This paper considers the management of the entire health care needs of a whole population through the provision of highly accessible, digital-first primary care in the English NHS (National Health Service). The NHS Long Term Plan states that all patients should have the right to choose fully digital-first primary care by March 31, 2024 [31]. Increased accessibility to primary care has been shown to reduce emergency department (ED) attendances [32], but little is known about the overall financial impact this service model will have on the health care system. The aim of this paper was to evaluate the impact that access to 24/7, highly accessible, digital-first primary care has on acute hospital spending.

## Methods

### Study Setting

Babylon GP at Hand is an NHS general practice in England, which is free at the point of need and provides full NHS primary care services under the General Medical Services contract to people living or working in London, Birmingham, and surrounding areas [33,34].

It was the first NHS general practice to adopt a fully digital-first model of primary care and has been operating across London in this way since November 2017. This means a member’s first and main point of contact is digital, with either a smartphone app or a web browser being used to access a virtual appointment. In-person services are available when required at 6 sites across London and 1 in Birmingham (2020).

Babylon GP at Hand is accessible for members day and night 365 days a year, with 80% of all appointments being digital (SG Winward, MD, unpublished data, April 1, 2019, to March 31, 2020). This is more than 3 times the “core hours” stipulated in the standard primary care contract (8 AM-6:30 PM Monday to Friday) [34]. Appointments are therefore available at a time and place convenient for members, and 40% of all appointments occur outside the contracted core hours. Access to medical advice and information is fast; 67% of virtual appointments are available within 2 hours of booking, and 81% of all appointments (including those in person) occur within 48 hours of booking (SG Winward, MD, unpublished data, April 1, 2019, to March 31, 2020); meanwhile, the national average of appointments occurring the same or next day is 49% [35]. Members also have access to a comprehensive suite of digital self-care technology, which can check symptoms; perform a digital health assessment; and monitor symptoms, observations, activity, and mood through the Babylon app.

All services are free for registered members, who must live or work within 40 minutes of a Babylon GP at Hand clinic to be eligible. If previously registered with another NHS practice, the members can switch to their registered GP practice to Babylon GP at Hand. The list size has grown from 3000 to over 100,000 members since November 2017, and the Babylon GP at Hand became the largest single practice in the United Kingdom in August 2020 [33].

### Data Sources

The commissioner spending on acute hospital care was compared between patients registered at Babylon GP at Hand and patients registered at other practices in the North West London Collaboration of Clinical Commissioning Groups for the NHS financial years (FYs) of April 1, 2018 to March 31, 2019 (FY18/19); and April 1, 2019, to March 31, 2020 (FY19/20).

Following a freedom of information (FOI) request, the total acute hospital spending for patients registered at each general practice in North West London Collaboration of Clinical Commissioning Groups was received and aggregated at the level of each practice (Multimedia Appendix 1). The data represented all costs incurred for patients registered at practices in the commissioning region at acute providers. This included acute and general hospital care, such as ED, inpatients, critical care, outpatients, and maternity services, including all associated costs, such as laboratory costs. It did not include mental health spending (eg, inpatient mental health admissions), community spending (eg, district nursing), or specialized services (eg, the treatment of rare cancers, genetic disorders, or complex medical or surgical conditions), as these services are not consistently provided by all acute trusts or are funded by national rather than local commissioners. Primary care prescription spending was not included in the FOI response, but medications prescribed in hospital were.

Practices were eligible for inclusion if they were active during FY18/19 or FY19/20. Spending was excluded that could not be associated with a patient population.

### Weighting the Populations

**Figure 1:**
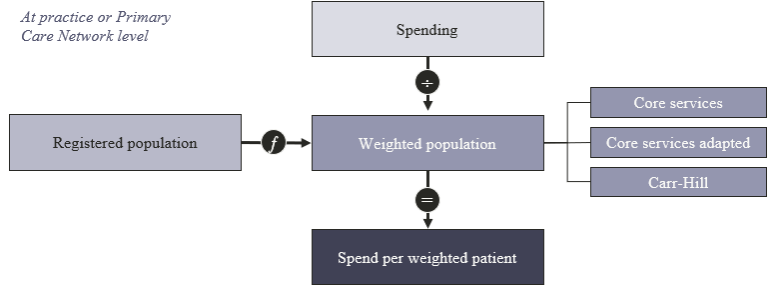
Overview of methodology.

Figure 1 shows an overview of the methodology used to compare spending; the total spending per practice is divided by the number of need-adjusted or “weighted” patients. Any differences in the spending per weighted patient between populations would therefore be for reasons other than health care need.

As the populations registered to each practice were not constant throughout the NHS financial year, an average registered population was calculated based on the practice population at the start of each quarter [33]. All further references to a practice’s registered population size are based on this averaged value.

The demographics and health needs of different populations can vary greatly, so each population must be adjusted before their spending can be compared. Existing NHS methodologies were replicated to achieve this. They exist to ensure public funding is distributed fairly by adjusting for characteristics known to impact health care spending, such as, age, sex, and deprivation (Table 1). This transformed the registered population for each practice into a weighted population. For example, if a population with certain characteristics was expected to incur twice the costs of another, it would have twice the number of weighted patients than the other.

Three methods were used to weight the populations, resulting in three weighted populations for each practice (Table 1) [36-39].

**Table 1:** Description of 3 methodologies used to weight patient populations to enable the comparison of spending.

Weighting	Description
Core services [36]	Around £80 billion of NHS^a^ funding per year is distributed to commissioners for “core services”. The core services formula is used to ensure fair distribution of this amount to populations with different characteristics. It has separate components that weight each practice’s population for their need for services, including general and acute, mental health, maternity, community care, and prescription needs. In addition, each population is adjusted for local factors: health care utilization, supply of health care services, pricing, unavoidable costs, unmet need (with premature mortality rate used as a proxy), local deprivation, and costs due to unavoidable smallness.
Core services adapted	Three adaptations were made to the core services methodology to better match the spending data received in the freedom of information request. First, as the spending data received did not include mental health, community, or prescription costs, these elements were removed from the formula. Second, the Babylon GP at Hand population was reweighted by the actual age and sex characteristics as opposed to the estimates provided in the core services file. As Babylon GP at Hand is a fast-growing practice, it was over 3 times the size of the estimation [37]. Third, the core services formula assumes that all Babylon GP at Hand patients live in Hammersmith and Fulham, whereas the majority live in other clinical commissioning groups. To improve the accuracy, the local components from each patient's home residence were used.
Carr-Hill [38,39]	The Carr-Hill weighting is used to distribute the global sum, the largest component of primary care funding. It adjusts the population based on drivers of need, including the consulting time recorded for patients with certain characteristics, local premature mortality rates, market forces (local costs), practices rurality index (though this has been phased out), and the number of nursing home patients registered to the practice.

^a^NHS: National Health Service.

### Cost Per Weighted Patient

The total spending for each practice was divided by its number of weighted patients, giving the spending per weighted patient for that practice. This was also performed at the level of the primary care networks, which are groups of practices that work collaboratively, totalling around 50,000 people each [40,41]. Primary care networks (groupings of general practices) are more similar in size to the Babylon GP at Hand practice than are other practices and hence are a more appropriate grouping to compare against.

### Adjustments for the Babylon GP at Hand Practice

To increase the accuracy of the calculation for Babylon GP at Hand, 3 adjustments were applied.

First, patients registered at Babylon GP at Hand’s Birmingham site were removed. As spending data were only received for hospitals in Greater London, no reciprocal adjustments were made to the spending. The Birmingham site opened in June 2019, so this only affected FY19/20.

Second, Babylon GP at Hand launched from an existing practice in July 2017. A cohort of patients who lived near to the Hammersmith and Fulham site continued to receive a traditional model of primary care from the existing provider. This population was removed to better assess the effects of Babylon GP at Hand model of care. As spend data were only received at a practice level, this group of patients was assigned the average weighting and average cost per patient for the Hammersmith and Fulham Clinical Commissioning Group to remove them from the Babylon GP at Hand practice totals.

Third, an independent review of Babylon GP at Hand, commissioned by NHS England, reported that patients who joined the practice were less likely to use certain hospital services in the 12 months prior to joining than were a matched population [42]. If this observation persisted after patient joining, this would imply that the Babylon GP at Hand population would incur lower costs even after adjustment for the characteristics of its populations. To conservatively account for this potential effect, the cost per weighted patient for Babylon GP at Hand was inflated. To determine the degree of inflation, population data, national reference costs, and attendance rates for each age and sex category were used to calculate the expected costs for the Babylon GP at Hand population [43,44]. The same calculation was then performed using the attendance rates modified by the findings in the independent review [42]. A 12% difference between the 2 represented the degree to which the actual cost would be lower than the expected cost, and hence the Babylon GP at Hand spending per weighted patient was increased by this factor.

### Adjustment for Inflation

Costs for FY18/19 were adjusted for inflation to be equivalent to FY19/20 values. The GDP deflator at market prices for the United Kingdom was used as produced by Her Majesty's Treasury and published by the Office of National Statistics [45].

### Data Analysis

Analysis was performed on SciPy package version 1.5.4 (Python) [46]. Shapiro-Wilk and Kolmogorov-Smirnov tests were used to determine if the data were normally distributed before a 1-sided, simple *z* test was performed to compare the spending per weighted patient between groups.

## Results

**Figure 2:**
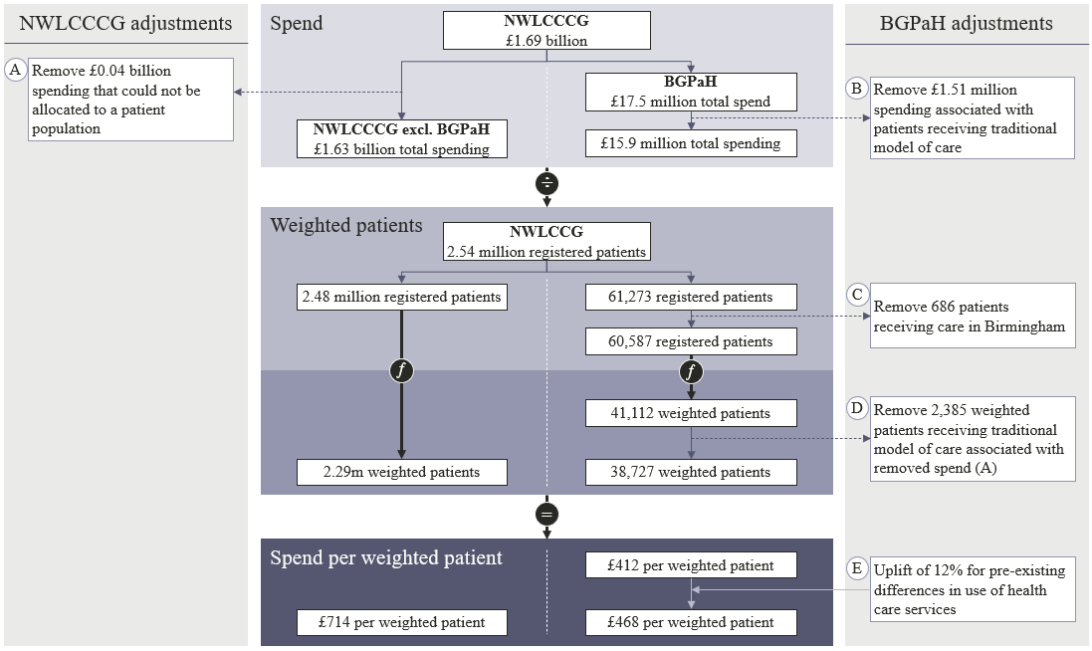
Flow chart demonstrating how the spending per weighted patient for the core services methodology was calculated for the financial year 2019-2020. BGPaH: Babylon GP at Hand practice; NWLCCCG: North West London Collaboration of Clinical Commissioning Groups.

Figure 2 follows the methodology outlined in Figure 1 to calculate the cost per weighted patient, with the core services methodology being used in FY19/20, including all adjustments.

### Spending

The total acute care spending returned in the FOI was £1.64 billion in FY18/19 and £1.69 billion in FY19/20 (a currency exchange rate of £1=US $1.38 is applicable), across 361 practices. An expenditure of £44.6 million (2.71%) in FY18/19 and £43.4 million (2.56%) in FY19/20 was excluded that was associated with 3 practices and an “Unknown Primary Care Network” in the FOI response, as the costs could not be attributed to a patient population (Figure 2A). The remaining spending was £1.6 billion in FY18/19 and £1.65 billion in FY19/20 across 358 practices including Babylon GP at Hand.

The spending for Babylon GP at Hand practice was £8.6 million in FY18/19 and £15.9 million in FY19/20,after the spending associated with patients receiving a traditional model of care (£1.59 million and £1.51 million, respectively) was removed (Figure 2B).

### Registered Population

The 358 practices had a total registered population of 2.43 million patients in FY18/19 and 2.54 million in FY19/20.

The registered population at Babylon GP at Hand was 32,393 and 61,273 patients for FY18/19 and FY19/20, respectively. There was total of 60,587 patients after 686 patients registered to the Birmingham site in FY19/20 were removed (Figure 2C). This population included 2563 and 2696 registered patients in FY18/19 and FY19/20, respectively, who received a traditional model of care. This population and the associated spending was removed (Figure 2D).

The remaining 357 practices in the region had an average of 6718 registered patients (range 235-21,688) in FY18/19 and 6943 (range 224-22,969) in FY19/20. There were 48 primary care networks (excluding the Babylon GP at Hand primary care network) in the North West London region, which had on average 49,682 registered patients (range 28,318-80,903) in FY18/19 and 51,358 (range 29,125-83,965) in FY19/20.

Compared with the North West London Collaboration of Clinical Commissioning Group population, the Babylon GP at Hand population was more concentrated in working age adults, had higher rates of employment, and experienced similar levels of deprivation (Table 2).

**Table 2:** Sociodemographic characteristics of the populations [33,47].

Sociodemographic indicators		FY18/19^a^		FY19/20^b^	
		BGPaH^c^ (n=32,394)	NWLCCCG^d^ (n=2,398,352)	BGPaH (n=60,587)	NWLCCCG (n=2,478,711)
**Population by age band, n (%)**					
	Female 0-19	729 (2.25)	255,803 (10.67)	987 (1.63)	262,433 (10.59)
	Female 20-39	12,221 (37.73)	425,275 (17.73)	24,040 (39.68)	437,143 (17.64)
	Female 40-59	1208 (3.73)	294,998 (12.3)	1881 (3.1)	306,834 (12.38)
	Female 60-79	205 (0.63)	154,813 (6.45)	244 (0.4)	160,536 (6.48)
	Female 80+	37 (0.11)	42,439 (1.77)	39 (0.06)	43,822 (1.77)
	Male 0-19	551 (1.7)	269,153 (11.22)	814 (1.34)	275,767 (11.13)
	Male 20-39	14,474 (44.68)	430,981 (17.97)	27,757 (45.81)	442,271 (17.84)
	Male 40-59	2675 (8.26)	348,327 (14.52)	4436 (7.32)	365,469 (14.74)
	Male 60-79	268 (0.83)	146,604 (6.11)	361 (0.6)	153,160 (6.18)
	Male 80+	26 (0.08)	29,959 (1.25)	29 (0.05)	31,276 (1.26)
Index of multiple deprivation^e^, percentile^f^		N/A^g^	N/A^g^	45th	52nd
**Employment status^h^ (%)**					
	Employed^i^	94.2	68.7	90.5	68.9
	Unemployed	0.1	5.3	4.0	5.3
	Other^j^	5.7	26.1	5.5	25.9

^a^FY18/19: financial years 2018-2019.

^b^FY19/20: financial years 2019-2020.

^c^BGPaH: Babylon GP at Hand practice.

^d^NWLCCCG: North West London Collaboration of Clinical Commissioning Groups.

^e^Available for 97.8% (349/357) of practices.

^f^With the 1st percentile representing the most deprived and the 100^th^ representing the least.

^g^N/A: not available.

^h^Absolute values were not published.

^i^Employed status included “Full-time paid work (30 hours or more each week)”; “Part-time paid work (under 30 hours each week)”; and “Full-time education at school, college or university”.

^j^Other included “Permanently sick or disabled,” “Fully retired from work,” “Looking after the family or home,” and “Doing something else”.

### Weighting of Patient Population

The average need index (the factor describing the size of the weighted population relative to the registered population) for Babylon GP at Hand was between 27.5% and 43.9% lower for the core services and the core services adapted weighting methodologies, respectively (Table 3). Smaller differences were observed in the Carr-Hill method.

**Table 3:** Average need indices for the 3 weighting methodologies for Babylon GP at Hand practice and the average of North West London Collaboration of Clinical Commissioning Groups for FY18/19 and FY19/20.

Weighting methodology		BGPaH^a^ need index	NWLCCCG^b^ need index	Difference^c^, absolute (%)
**Core services**				
	FY18/19^d^	0.66	0.93	0.26 (28.4)
	FY19/20^e^	0.67	0.92	0.25 (27.5)
**Core services adapted**				
	FY18/19	0.50	0.88	0.39 (43.7)
	FY19/20	0.49	0.88	0.39 (43.9)
**Carr-Hill**				
	FY18/19	1.00	0.93	–0.08 (–8.2)
	FY19/20	0.90	0.93	0.03 (3.2)

^a^BGPaH: Babylon GP at Hand practice.

^b^NWLCCCG: North West London Collaboration of Clinical Commissioning Groups.

^c^Numbers may not sum due to rounding.

^d^FY18/19: financial years 2018-2019.

^e^FY19/20: financial years 2019-2020.

### Cost Per Weighted Patient

Before statistical analysis, the cost per weighted patient for Babylon GP at Hand was increased by 12% (Figure 2E) to correct for lower than expected activity rates reported in an independent review of the practice [42].

The 1-sided, simple *z* test was performed on a primary care network level, where the spending per weighted patient was normally distributed. A significantly lower cost per weighted patient for Babylon GP at Hand was observed for all weighting methodologies across both years. This was between 12.4% (£93) to 54.4% (£389) lower in FY18/19 and 15.2% (£114) to 50.9% (£362) lower in FY19/20 (Table 4).

Practice-level data were not normally distributed, there was a high number of outliers, and the Babylon GP at Hand practice was not of a comparable size; thus, the 1-sided, simple *z* test was not performed. The Babylon GP at Hand practice’s percentile among the other 357 practices is shown Table 5.

**Table 4:** Summary of cost per weighted patient for Babylon GP at Hand compared with the North West London Collaboration of Clinical Commissioning Groups average, including absolute and percentage differences and 1-sided, simple *z* test results for FY18/19 and FY19/20.

Weighting methodology			BGPaH^a^ cost (£) per weighted patient, £		NWLCCCG^b^ cost (£) per weighted patient, £		Difference in cost^c^ (£), amount (%)		*P* value
**Core services**									
	FY18/19^de^	492		715		–223 (–31.2)		<.001	
	FY19/20^f^	468		714		–246 (–34.5)		<.001	
**Core services adapted**									
	FY18/19^e^	656		748		–93 (–12.4)		.047	
	FY19/20	635		749		–114 (-15.2)		.006	
**Carr-Hill**									
	FY18/19^e^	325		714		–389 (–54.4)		<.001	
	FY19/20	349		711		–362 (–50.9)		<.001	

^a^BGPaH: Babylon GP at Hand.

^b^NWLCCCG: North West London Collaboration of Clinical Commissioning Group average, excluding Babylon GP at Hand.

^c^Numbers may not sum due to rounding.

^d^FY18/19: financial years 2018-2019.

^e^Adjusted for inflation to be comparable to FY19/20 costs.

^f^FY19/20: financial years 2019-2020.

**Table 5:** Percentile rank of Babylon GP at Hand practice among all practices in North West Central London Collaboration of Clinical Commissioning Groups.

Weighting methodology		Babylon GP at Hand, percentile^a^
**Core services**		
	FY18/19^b^	3rd
	FY19/20^c^	3rd
**Core services adapted**		
	FY18/19	15th
	FY19/20	9th
**Carr-Hill**		
	FY18/19	2nd
	FY19/20	2nd

^a^With the 1^st^ percentile representing the lowest spending per weighted patient and the 100^th^ representing the highest.

^b^FY18/19: financial years 2018-2019.

^c^FY19/20: financial years 2019-2020.

A summary of the total acute spending, registered populations, need indices, weighted populations, and cost per weighted patient for each practice and primary care network can be found in Multimedia Appendix 2.

## Discussion

### Principal Results

This paper is the first to show that an association between a highly accessible, 24/7, digital-first model of primary care and significantly lower acute hospital costs. This was observed over 2 consecutive years and across all 3 methodologies used to adjust for health care need. The spending per weighted patient for Babylon GP at Hand practice was 12%, 31%, and 54% (£93, *P*=.047; £223, *P*<.001; and £389, *P*<.001) lower than the regional average in FY18/19 for the core services adapted, core services, and Carr-Hill weighting methodologies, respectively. In FY19/20, it was 15%, 35%, and 51% (£114, *P*=.006; £246, *P*<.001; and £362, *P*<.001) lower. This represented a lower total spending for the Babylon GP at Hand population in FY18/19 of £1.37 million, £4.40 million, and £11.6 million for the core services adapted, core services, and Carr-Hill weighting methodologies, respectively. In FY19/20, the equivalent figures were £3.26 million, £9.54 million, and £18.8 million, respectively.

The reduction in hospital care costs observed is likely to be much greater than the additional cost of delivering 24/7, digital-first primary care. In FY19/20, the Babylon GP at Hand practice delivered 23% more appointments per Carr-Hill weighted patient than the national average [35]. Even if primary care costs grew linearly with the number of appointments, this would translate to additional costs of £36 (based on an average funding per patient of £155 in FY19/20 [48] and assuming that primary care funding equals the costs of provision). Even after these additional costs were accounted for, the savings in FY19/20 would still be between £78 and £326 per weighted patient. Furthermore, any additional digital-first primary care costs are borne by the provider (Babylon GP at Hand) and not by the NHS, as NHS primary care practice payments are capitated rather than activity based. The full acute cost savings therefore accrue to the NHS.

### Limitations

First, the main limitation is that patient-level data were not available; therefore, it was not possible to examine the causal factors behind the lower costs observed for patients receiving 24/7, digital-first, primary care. Second, given that patients chose which practice to join, there might have been a degree of self-selection that was not corrected for by the weighting formulae used. However, adjustments, such as prior use of health care services by Babylon GP at Hand members, were made for known differences. This was conservative and acted to increase the cost per weighted person for the Babylon GP at Hand practice, suggesting that the cost savings may be greater than those shown. Further work is needed to access patient-level data, which could explain in which areas savings are made, eliminate self-selection bias, and reduce the need for adjustments.

The wider applicability of the findings is limited in part by the registered population of Babylon GP at Hand and by the spending categories returned in the FOI request. The population of Babylon GP at Hand is concentrated in working age adults, 95.9% (58,113/60,587) of patients in FY19/20 were between 20 to 59 years old compared to 62.6% (1,551,717/2,478,709) in the rest of the region. This could partially explain the higher percentage of employment observed in the Babylon GP at Hand population. The weighting formulae adjusted for the population differences, as evidenced by the Core Service Adapted need index in FY19/20 being 43.9% lower for Babylon GP at Hand than the regional average. However, interpretation of the findings is limited for other age ranges. The spending data returned in the FOI request did not include mental health, community, or primary care prescription spending, which represented 36% of the total budget for the Clinical Commissioning Group core services in FY19/20 [36]. Resultantly, the effect of 24/7 digital-first health care on this portion of spending has not been determined. However, there are reasons to believe that the same effect would be observed in these categories, as has been shown elsewhere [49].

This study is focused on acute hospital spending but did not assess the quality and therefore health care value. Assessing the quality of primary care is difficult given its broadness and the lack of robust quality metrics. However, during the period of investigation, Babylon GP at Hand practice was rated “Good” by the Care Quality Commission; scored 92% and 96% in all available Quality Outcome Framework points in FY18/19 and FY19/20 [50], respectively; and 94.4% (146,077/154,738) of patient ratings for clinical consultations were 4 or 5 stars out of 5 during the study period (SG Winward, MD, unpublished data, April 1, 2018, to March 31, 2020).

The Carr-Hill weighting methodology factors in demographics and other drivers of need, but its purpose is to determine primary care funding rather than acute care spending. This weighting approach was recommended in the FOI response and hence it was included but is not considered as robust as the core services adapted and core services methodologies. Therefore, the central finding of this paper is a 15%-35% lower spending per weighted patient for members of Babylon GP at Hand in FY19/20.

The accuracy of the analysis in this paper is contingent on the quality and reliability of the NHS data that were provided in the FOI request (Multimedia Appendix 1).

### Comparison With Prior Work

To our knowledge, this study is the first to assess the impact that a highly accessible, digital-first model of primary care has on acute hospital spending. Although the findings cannot infer causality, they are consistent with those of other publications. These include, for example, a link between accessible primary care and reduced demand for other services [32]; and an NHS England commissioned report on Babylon GP at Hand, which found patients were significantly less likely to attend ED and outpatient appointments than was a control population [42]. The authors of the report have since called for more evidence to be obtained on the sustainability of such services, as this paper seeks to provide [51].

The paper is aligned with the majority of the literature in showing that digital health solutions can reduce costs [23-25,27]. It adds insight in two areas where research is sparse. First, it is an assessment of a digital-first model of care for a wide range of conditions as opposed to a digital tool in a single condition; second, it quantifies cost savings from the perspective of the health system [19]. The results are particularly important in the context of the national direction of travel laid out in the NHS Long Term Plan, which states that all patients must be able to access digital-first primary care by the end of the 2024 financial year [31].

Several areas requiring further research have been identified. To increase confidence in the conclusions, assessment of patient-level data over all spending categories (ie, mental health, prescriptions, and community and acute hospital spending) during the same period is required. Further research is also needed to fully assess the impact of the model of care on quality outcomes. The effectiveness of telehealth solutions has been shown to be linked to the provider [27], so further work with alternate providers is required to establish if the observed benefits are uniform.

### Conclusions

This paper has demonstrated that highly accessible, 24/7, digital-first primary care was associated with lower acute hospital spending for a health system. This effect was sustained over a 2-year period, during which the population under investigation doubled in size, demonstrating that the effect is scalable. Further work using patient-level data is needed to be able to generalize these findings to a wider demographic of patients and to understand the efficacy of digital-first primary care across different populations of patients.

## Appendix

Multimedia Appendix 1 Freedom of Information (FOI) request and response.

Multimedia Appendix 2 Summary of results.

## Abbreviations

ED: emergency department
FOI: freedom of information
FY: financial year
GDP: gross domestic product
NHS: National Health Service (of the United Kingdom)
